# The phlebotomine sand flies fauna in Parque Estadual do Rio Doce, Minas Gerais, Brazil

**DOI:** 10.1186/s13071-015-1227-1

**Published:** 2015-12-02

**Authors:** Cristian Ferreira de Souza, Reginaldo Peçanha Brazil, Paula Dias Bevilacqua, Jose Dilermando Andrade Filho

**Affiliations:** Programa de Pós-Graduação em Biologia Parasitária, Instituto Oswaldo Cruz/Fiocruz, Avenida Brasil, 4365 - Manguinhos, CEP: 21040-900 Rio de Janeiro-RJ, Brazil; Laboratório de Doenças Parasitarias, Instituto Oswaldo Cruz/Fiocruz, Avenida Brasil, 4365 - Manguinhos, CEP: 21040-900 Rio de Janeiro-RJ, Brazil; Departamento de Veterinária, Universidade Federal de Viçosa, Avenida Peter Henry Rolfs, s/n - Campus Universitário, CEP: 36570-000 Viçosa-MG, Brazil; Centro de Referência Nacional e Internacional para Flebotomíneos-Centro de Pesquisas René Rachou/Fiocruz, Avenida Augusto de Lima, 1715 - Barro Preto, CP1743, CEP: 30190-002 Belo Horizonte-MG, Brazil

**Keywords:** Phlebotominae, Leishmaniasis, Rain forest, Brazil

## Abstract

**Background:**

Phlebotomine sand flies are dipterans of the family Psychodidae. They are very important to veterinary medicine because some species are vectors of infective forms of *Leishmania* spp., the etiological agents of leishmaniasis. The Parque Estadual do Rio Doce is located in an area with constant reports of cases of leishmaniasis. In order to better understanding the phlebotamine sand fly fauna of the park, the present work was undertaken with the goal of analyzing phlebotomine sand flies collected there, verifying their seasonality and correlating their presence with forest and/or anthropic areas.

**Methods:**

To analyze the fauna of phlebotomine sand flies, HP-type, model CDC light traps were distributed along the Juquita trail of PERD. Twelve traps were installed between September 2012 and February 2014, and captured specimens were identified to species.

**Results and discussions:**

A total of 1993 phlebotomine sand flies of 30 species were captured. The most abundant species were *Pressatia choti*, *Psychodopygus davisi* and *Nyssomyia intermedia*. The high number of *Nyssomyia intermedia* captured drew attention because they are considered one of the vectors of the infective *Leishmania braziliensis* present at PERD. No seasonality was observed in the occurrence of phlebotomine sand flies captured at PERD. The number of captured specimens of vector species, and the distance of traps from the forest boarder, were negatively correlated, showing that these vectors (*Nyssomyia intermedia*, *Nyssomyia whitmani* and *Migonemyia migonei)* were less common inside the forest area and that attention should be drawn to other potential vector species in the forest.

**Conclusion:**

These results can contribute to leishmaniasis prevention strategies directed at the visitors and professionals at or near PERD. The finding of the presence of *Leishmania* vectors in the park area must be given attention, since disease transmission can threaten people who visit PERD and its surroundings. Therefore, information on the prevention of leishmaniasis needs to be provided to all people who go there.

## Background

Phlebotomine sand flies (Diptera: Psychodidae: Phlebotominae) are of particular importance to veterinary medicine because some species are vectors of the infective form of *Leishmania* spp., etiological agents of leishmaniasis. Leishmaniasis is a group of diseases classified as visceral leishmaniasis (VL), which presents a serious form of the disease affecting the internal organs, and tegumentary leishmaniasis (TL), a more common form that causes ulcers of the skin and mucosal destruction of upper respiratory tract. Leishmaniases are endemic in many regions of the world, including Brazil [1, 2], and current research is looking for information regarding their urban cycle, focusing on their adaptation to urban areas [3, 4].

According to data from the National System of Notification, between the years 2007 and 2013, there was a yearly average of 3674 cases of VL and 22,601 of TL. During the same period, the state of Minas Gerais, Brazil, reported 3321 cases of VL and 9721 cases of TL, placing it among the top five Brazilian states in terms of the number of reported cases of leishmaniases [5].

Originally transmission of leishmaniasis was primarily associated with rural and wild areas. However, with changes in behavior and habitat use of vectors and reservoirs, and their adaptation to the anthropic environment, research has begun to investigate domiciliary and peridomiciliary areas [6–8]. Nonetheless, there remains a need for new information about leishmaniasis in wild areas since these areas are constantly being used for scenic beauty, ecological sports, tourist attractions, and education and research.

Parque Estadual do Rio Doce (PERD) is an area that receives a large number of visitors throughout the year, not only for research and tourism, but also for teaching students from nearby schools.

According to unpublished information from PERD, there are constant reports of cases of leishmaniasis among the workers that live in houses located in the park; these cases are reported and analyzed as cases from neighboring cities, such as Timóteo, Ipatinga and Marlieria.

Knowing the composition of the phlebotomine sand fly fauna that exists in PERD is vital to a better understanding the transmission cycle of leishmaniasis in and around the park and to identify possible vectors. Analyzing the seasonal occurrence of species of phlebotomine sand flies is also important and has been explored by different studies, which have found that the climatic variables related to their occurrence are, in general, temperature, rainfall and air relative humidity [9–14]. These variables have been used previously to explain the behavior of populations of phlebotomine sand flies and, consequently, variation in the occurrence of human cases. This can also be of importance to find a better attitude for the control of leishmaniasis in areas of risk.

The goal of the present study was to investigate the phlebotomine sand fly fauna of Parque Estadual do Rio Doce. More specifically, the seasonal occurrence of phlebotomine sand flies will be analyzed, as will the location of vectors relative to the forest boarder, in order to better understand the dynamics of leishmaniasis transmission in the area.

## Methods

Parque Estadual do Rio Doce (PERD) was the first Conservation Unit to be established in the state of Minas Gerais, and is located among the municipalities of Timóteo, Marliéria and Dionísio. It remains one of the largest conservation areas of Atlantic Rainforest in the country with 35,970 hectares, and is the third largest flooded area in Brazil after the Amazon and the Pantanal [15].

Daily, PERD receives tourists, researchers and teaching institutes in order to promote environmental education, ecologic tourism and biodiversity research. Currently, the park contains two trails that are constantly used for these activities: the Vinhático trail and the Juquita trail. The Juquita trail is located in the part of the park that lies within the municipality of Timóteo. Juquita trail was chosen for this study because of its proximity to a knwon endemic area of TL and because it receives frequent visits [15].

**Fig. 1:**
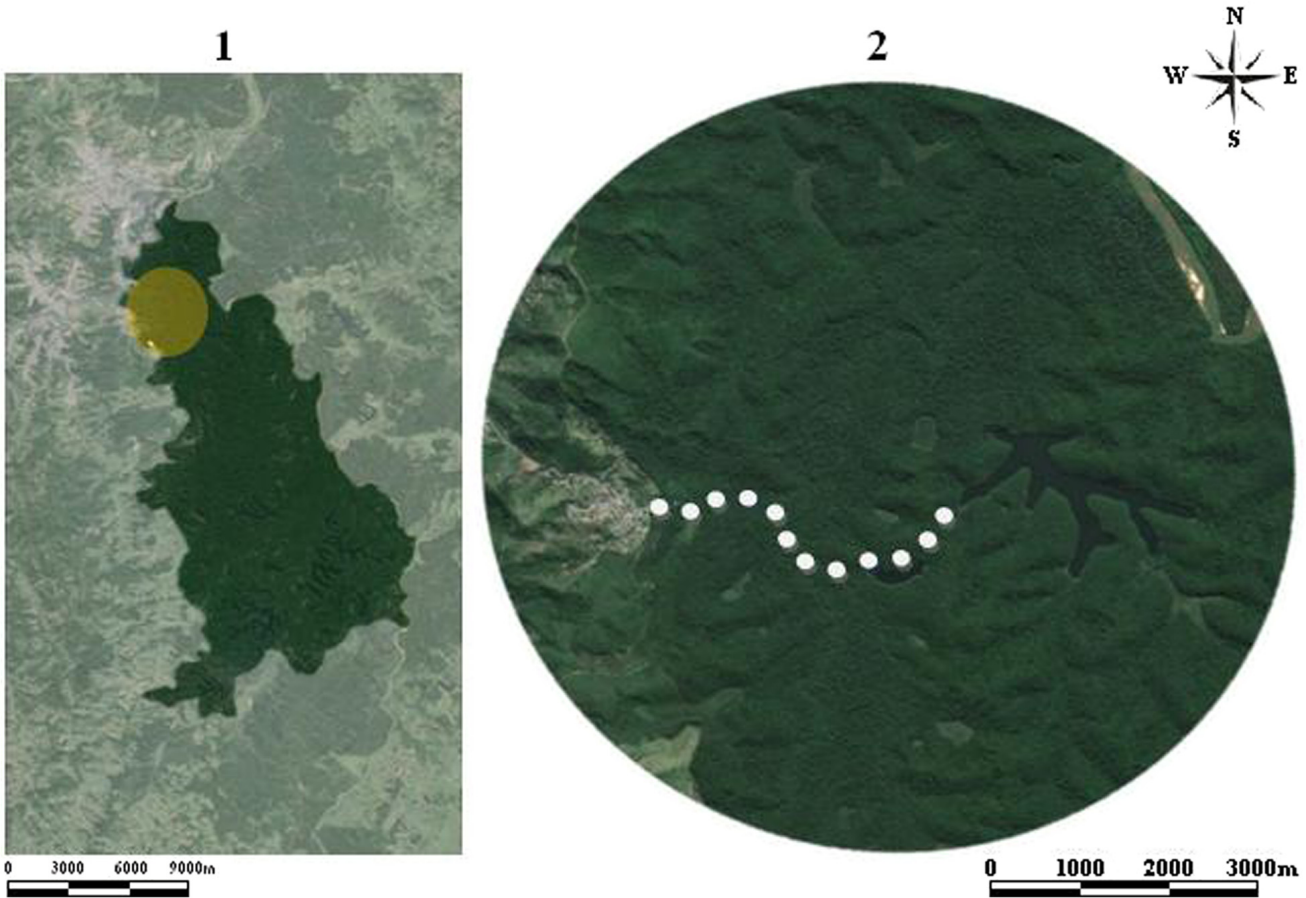
(1) Map of Parque Estadual do Rio Doce (PERD); (2) (○) White dots indicating the distribution of light traps along Juquita trail at PERD, Minas Gerais, Brazil. Source: https://maps.google.com.br/

Phlebotomine sand flies were collected using HP-type, CDC model light traps distributed along the Juquita trail, which starts at the boarder of the park and the urban area of the municipality of Timóteo, and continues into the forest for 3300 m. The light traps were placed at sampling points along the trail at 300-meter intervals. The points were distributed in the following manner: Point 1–0 meter (m) (begin of trail); Point 2–300 m; Point 3–600 m; Point 4–900 m; Point 5–1200 m; Point 6–1500 m; Point 7–1800 m; Point 8–2100 m; Point 9–2400 m; Point 10–2700 m; Point 11–3000 m; Point 12–3300 m (Fig. 1). Sampling was performed from September 2012 to February 2014 with the traps being active for 48 h consecutively once each month for a total of 864 h of capture effort.

Captured male and female flies were slide-mounted and identified according to the classification of Galati [16]; the females were subjected to molecular review after identification. Species names and abbreviations follow the proposal of Marcondes [17]. Data were compiled in tables, and evaluated through the calculation of the proportion and prevalence of phlebotomine sand flies according to species and sex; sex ratios were compared using the Z test.

Seasonal behavior was analyzed using the proportional distribution of phlebotomine sand flies across monthly captures and the climatic variables of temperature (°C), rainfall (%) and humidity (mm). Descriptive analyses (mean and median) were performed on the climatic variables. After testing for normality, Spearman correlation coefficients were calculated between the proportions of captured phlebotomine sand flies and the climatic variables. Climatic variable data were obtained from the National Institute of Meteorology [18] for the sampling period of September 2012 to February 2014.

Temperature and humidity were analyzed using the mean and median, whereas rainfall was analyzed using only the mean.

Three species of known vectors of *Leishmania* spp. (*Nyssomyia intermedia, Nyssomyia whitmani, Migonemyia migonei*), were selected to document their distribution along the trail. These analyses were done, after testing for normality, by calculating the Spearman correlation coefficients between the proportions of phlebotomine sand flies and trap location. These analyses were carried out using Microsoft® Office Excel 2012 and STATA®12 programs and a 5 % (α = 0.05) significance level was employed. Collecting of sand flies within PERD was performed under a permanent license to collect zoological material (N. 16,237-2), granted by the Environment Ministry.

## Results and discussion

A total of 1993 specimens of phlebotomine sand flies belonging to 30 species were captured (Table 1). The most abundant species were *Pressatia choti* (19.12 %)*, Psychodopygus davisi* (15.65 %) and *Nyssomyia intermedia* (11.34 %). The most abundant species, *Pressatia choti*, should not be of great concern because it is not involved in the transmission of *Leishmania* ssp. However, the same cannot be stated about *Ps. davisi,* which was has been associated with the transmission of *Leishmania* (*Viannia*) *naiffi*, in the Amazon region [19], and later in the state of Roraima [20]. This species of *Leishmania* is responsible for cutaneous leishmaniasis in the Amazon region [20], and draws attention to the possibility of new vectors in the transmission cycle of leishmaniasis at PERD.

**Table 1:** Frequency of species of phlebotomine sand flies captured, according to sex, Parque Estadual do Rio Doce, Minas Gerais, Brazil, from September 2012 to February 2014

Species	Male	Percent	Female	Percent	Total	Percent
*Lutzomyia* sp.	0	0.00	6	0.49	6	0.30
*Brumptomyia* sp.	0	0.00	42	3.45	42	2.11
*Br. avellari*	54	6.96	0	0.00	54	2.71
*Br. nitzulescui*	40	5.15	0	0.00	40	2.01
*Pa. aragaoi*	0	0.00	2	0.16	2	0.10
*Pi. bianchigalatiae*	0	0.00	1	0.08	1	0.05
*Ps. geniculatus*	0	0.00	4	0.33	4	0.20
*Mi. borgmeirai*	20	2.58	1	0.08	21	1.05
*Mi. capixaba*	0	0.00	45	3.70	45	2.26
*Ps. carreirai*	28	3.61	143	11.75	171	8.58
*Pr. choti*	198	25.52	183	15.04	381	19.12
*Ev. cortelezzii*	0	0.00	1	0.08	1	0.05
*Ev. costalimai*	1	0.13	0	0.00	1	0.05
*Ps. davisi*	18	2.32	294	24.16	312	15.65
*Ev. edwardsi*	0	0.00	4	0.33	4	0.20
*Pi. fischeri*	3	0.39	41	3.37	44	2.21
*Pa. lanei*	0	0.00	7	0.58	7	0.35
*Ny. intermedia*	141	18.17	85	6.98	226	11.34
*Ty. longispina*	139	17.91	67	5.51	206	10.34
*Pa. lutziana*	0	0.00	7	0.58	7	0.35
*Mg. migonei*	16	2.06	48	3.94	64	3.21
*Pi. misionensis*	0	0.00	5	0.41	5	0.25
*Mi. oswaldoi*	0	0.00	3	0.25	3	0.15
*Pi. pessoai*	0	0.00	3	0.25	3	0.15
*Pa. pascalei*	97	12.50	59	4.85	156	7.83
*Mi. quinquefer*	1	0.13	16	1.31	17	0.85
*Ev. sallesi*	0	0.00	3	0.25	3	0.15
*Pa. shannoni*	0	0.00	2	0.16	2	0.10
*Sc. sordellii*	2	0.26	20	1.64	22	1.10
*Ev. termitophila*	1	0.13	4	0.33	5	0.25
*Ev. tupynambai*	1	0.13	46	3.78	47	2.36
*Ny. whitmani*	16	2.06	75	6.16	91	4.57
Total (%)^a^	776(38.94)	100.00	1217(61.06)	100.00	1993	100.00

^a^Comparison between the proportions of captured phlebotomine sand flies according to sex: z = 13.97; *p* <0,0001

*Ny. intermedia* is a species that draws attention because it is one of the known vectors of *Leishmania braziliensis* [21, 22] in the rain forest, and it is a known vector of leishmaniasis. The large number of captured individuals of *Ny. intermedia* indicates that this species is likely one of the main vectors of tegumentary leishmaniasis in and around PERD.

In addition to *Ny. intermedia*, two other species that are recognized as vectors were captured at PERD, *Ny. whitmani* (4.57 %) and *Mi. migonei* (3.21 %). Despite not being predominant, their presence generates concern since they are known vectors of *Leishmania* ssp. [1, 23].

A strong negative correlation was identified between vector species and trap distance (into the forest) from the forest border (Table 2); as trap distance from the forest border increased, the number of captured known vectors (*Ny. intermedia, Ny. whitmani and Mi. migonei*) decreased. For example, 54.36 % of the known vector species of phlebotomine sand flies were captured in the first 300 m of the trail. It should be noted that that the boarder of the forest (and the beginning of the Juquita trail) is on the edge of the urban area of Timóteo, a known endemic area of tegumentary leishmaniasis [4], an anthropic area, and an area with a strong presence of domestic animals and crops, all contributing to the maintenance and occurrence of these vectors.

**Table 2:** Spearman correlation between captured phlebotomine sand flies and distance inside forest from the forest border, Parque Estadual do Rio Doce, Minas Gerais, Brazil, from September 2012 to February 2014

Collect Place (m)^ab^	Consecrated phlebotomine sand flies vectors^b^	Percent	Other phlebotomine sand flies species	Percent	Total	Percent
0	137	36.15	65	4.03	202	10.14
300	69	18.21	56	3.47	125	6.27
600	15	3.96	94	5.82	109	5.47
900	25	6.60	158	9.79	183	9.18
1200	26	6.86	270	16.73	296	14.85
1500	23	6.07	120	7.43	143	7.18
1800	21	5.54	257	15.92	278	13.95
2100	6	1.58	106	6.57	112	5.62
2400	7	1.85	145	8.98	152	7.63
2700	37	9.76	205	12.70	242	12.14
3000	3	0.79	35	2.17	38	1.91
3300	10	2.64	103	6.38	113	5.67
Total (%)	379(19.02)	100.00	1.614(80.98)	100.00	1993	100.00

^a^places where the traps were installed. distance from the beginning of the Juquita trail (forest boarder) into the forest. ^b^Correlation between the collect location and the number of phlebotomines considered. according to the literature. leishmaniasis vectors: *n* = 12; ρ = −0.6643. *P* > 0.0185. Documented phlebotomine sand flies vectors = *Nyssomyia intermedia. Nyssomyia whitmani. Migonemyia migonei*

**Table 3:** Correlation between the number of captured phlebotomine sand flies and the mean and median of climatic variables during the period of collection in the Parque Estadual do Rio Doce, Minas Gerais, Brazil. from September 2012 to February 2014

Period		(%)	Rainfall (mm)				Humidity (%)				Temperature (°C)			
			μ (%)	Med	SD	CV(%)	μ (%)	Med	SD	CV(%)	μ (%)	Med	SD	CV(%)
September/2012	19	0.95	0.0	0.0	0.0	0.0	60.1	60.0	15.1	25.12	20.4	19.6	3.3	16.18
October /2012	71	3.56	0.0	0.0	0.0	0.0	50.6	48.0	14.8	29.25	21.7	21.7	3.3	15.21
November /2012	51	2.56	0.0	0.0	0.1	0.0	78.7	79.0	9.1	11.56	24.4	24.3	2.4	9.84
December /2012	180	9.03	0.0	0.0	0.0	0.0	66.3	67.0	12.7	19.16	24.6	24.1	2.5	10.16
January /2012^a^	267	13.40	-	-	-	-	-	-	-	-	-	-	-	-
February /2012	163	8.18	0.1	0.0	0.3	300.00	64.5	66.0	12.4	19.22	25.2	24.7	2.7	10.71
March/2012	238	11.94	0.0	0.0	0.0	0.0	75.8	76.0	9.0	11.87	25.3	25.2	2.6	10.28
April/2012	208	10.44	1.4	0.0	3.8	271.42	89.3	91.0	5.6	6.27	22.1	22.0	1.5	6.79
May /2012	169	8.48	0.0	0.0	0.0	0.0	78.0	82.0	11.4	14.62	21.0	20.0	2.7	12.86
June/2012	74	3.71	0.0	0.0	0.0	0.0	79.2	82.0	12.1	15.28	19.3	18.7	2.7	13.99
July /2012	133	6.67	0.0	0.0	0.0	0.0	76.4	80.0	11.2	14.66	21.0	20.2	2.8	13.33
August /2012	68	3.41	0.0	0.0	0.0	0.0	61.6	60.0	14.7	23.86	21.6	20.5	3.5	16.20
September /2012	49	2.46	0.0	0.0	0.0	0.0	62.0	63.0	15.5	25.00	22.1	21.0	3.2	14.48
October /2012	8	0.40	0.8	0.0	1.0	125.00	94.1	95.0	3.6	3.83	15.7	15.5	0.9	5.73
November/2012	71	3.56	1.9	0.0	3.6	189.47	80.3	86.0	16.3	20.30	21.3	20.4	2.9	13.62
December/2012	173	8.68	0.7	0.0	2.2	314.28	74.0	73.0	16.1	21.76	25.7	24.6	4.0	15.56
Janeiro/2012	32	1.61	0.0	0.0	0.0	0.0	64.0	61.0	12.1	18.91	25.9	25.7	3.5	13.51
February/2012	19	0.95	0.0	0.0	0.0	0.0	60.3	59.0	15.1	25.04	25.3	24.2	3.3	13.04
Total	1.993	100.00	-	-	-	-	-	-	-	-	-	-	-	-

Note ^a^Period of deactivation of the meteorological station. μ - Mean; Med - Median; SD - Standard Deviation; CV - Coefficient of variation. Average rainfall: *n* = 17; ρ = 0.1530; *P* = 0.5577. Average humidity: *n* = 17; ρ = 0.2859; *P* = 0.2660. Average humidity: *n* = 17; ρ = 0.3163; *P* = 0.2161 Average temperature: *n* = 17; ρ = 0.2551; *P* = 0.3231. Average temperature: *n* = 17; ρ = 0.3043; *P* = 0.2350

The perimeter of the area of high occurrence of vectors, the first 300 m of the Juquita trail, generates concern because it is an area that receives intense visitation by researches, students, teachers, ecotourists, workers and so on, and therefore puts the health of many people at risk. The correlation data also suggest the possibility that other vector species are participating in the transmission cycle of leishmaniasis inside the forest, since the number of known vector species decreases further into the forest, by *Leishmania* transmission does not. These findings emphasize the importance for further study of natural infection among the phlebotomines at PERD.

A seasonal pattern (Table 3) of occurrence of phelbotomine sand flies was not found, as reported by other studies [4, 24]. Contrary to the findings of the present study, seasonal behavior patterns have been documented previously [4, 15, 25], which may indicate, in this case, punctual transmission of *Leishmania* in determined moments of the year. It is interesting to note that the sampling sites in an area of rain forest may be located in microhabitats that can change, mainly regarding temperature and humidity, thus altering population dynamics [11]. So although the present study did not find a pattern of seasonal behavior, it would be interesting to perform a more detailed study involving seasonal patterns of microhabitats in order to characterize more precisely the seasonal behavior of phlebotomines sand flies.

## Conclusion

The PERD possesses a high diversity of species of phlebotomine sand flies. The present study allowed us to suggest that the transmission cycle of *Leishmania* spp. is occurring closer to the forest boarder. This claim is based on the presence of known vectors species in the first 300 m of the Juquita trail, with a predominance of *Ny. intermedia*. This observation leads to the possibility that the transmission cycle of *Leishmania* is occurring inside the forest with the participation of other species of phlebotomine sand flies, since a correlation between the number of known vectors and the distance from the border of the forest is strongly negative.

Analyses of climatic variables did not reveal any pattern of seasonal behavior suggesting the absence of seasonality in the behavior of phlebotomine sand flies at PERD, although differences in microhabitats may exist, they are not measured by the meteorological stations. The results of the present study provide additional information that should be helpful in the adoption of preventive measures inside PERD, which need to include educating all people who use the park.
